# Optimising the collection of female genital tract fluid for cytokine analysis in pregnant women

**DOI:** 10.1016/j.jim.2018.03.014

**Published:** 2018-07

**Authors:** C.S. Short, R. Quinlan, P. Bennett, R.J. Shattock, G.P. Taylor

**Affiliations:** a Imperial College London, United Kingdom; b Imperial College Healthcare NHS Trust, United Kingdom

**Keywords:** Female genital tract fluid, Cervicovaginal fluid, Pregnancy, Cytokines, Menstrual cup, Ophthalmic sponge

## Abstract

**Introduction:**

To better understand the immunology of pregnancy, study of female genital tract fluid (FGF) is desirable. However the optimum method of collection of FGF in pregnant women for immunological methods, specifically cytokine measurement, is unknown.

**Methods:**

A prospective study of HIV-uninfected pregnant women comparing two methods of FGF collection: polyvinyl acetal sponge collection of cervical fluid (CF) and menstrual cup collection of cervicovaginal fluid (CVF). Samples were collected at 3 time points across the second and third trimesters: 14–21, 22–25 and 26–31 weeks. Multiplex chemi-luminescent assays were used to measure: IFN-γ, IL-1β, IL-2, IL-4, IL-6, IL-8, IL-10, IL-12, IL-13 and TNF-α. Optimal methodology for cytokine normalisation (sample weight, volume and total protein) was explored.

**Results:**

All cytokines were measurable in both fluid types. IL-1β, IL-8 and IL-6 were detected at the highest concentrations (ranking order CF > CVF > plasma). CVF collection was simpler, provided the largest volume of sample (median 0.5 g) with the potential for undiluted usage, and allowed for self-insertion. CF cytokine concentrations were intrinsically associated with sample weight and protein concentration however CVF cytokines were independent of these.

**Conclusion:**

Both methods of collection are robust for measurement of FGF cytokines during pregnancy. We recommend CVF collection using a menstrual cup as a viable option in pregnant women for high dimensional biological techniques.

## Highlights

• PVA sponges and MCs are robust methods for measuring FGF cytokines in pregnancy.
• MCs enable collection of large undiluted CVF volumes for high dimensional assays.
• CVF (not CF) cytokine concentrations are largely independent of sample weight or protein.
• Self-insertion and short collection time of MCs is an attractive option to women.
• Cytokine concentrations are higher in sponge samples reflecting the CF immune site.

## 1 Introduction

The study of female genital tract fluid (FGF) to further understand the molecular aetiology of obstetric conditions (e.g. preterm birth) and sexually transmitted infections (e.g. Human Papilloma Virus (HPV) and Human Immunodeficiency Virus (HIV)) is an expanding field (Wei et al., 2010; Castle et al., 2004; Nguyen et al., 2005; Dezzutti et al., 2011; Archary et al., 2015). FGF comprises of cervical mucous/fluid (CF) and vaginal secretions or a combination of the two (cervicovaginal fluid (CVF)). An advantage of this fluid type is that, in addition to studying the local vaginal immune compartment, it can also provide information about the upstream uterine and cervical environment (Dasari et al., 2007; Zegels et al., 2010). Another benefit is that it is easily obtained without the need for invasive procedures. This fluid has been used for a multitude of assays including the characterization of cytokines and other immune mediators, biomarker discovery through high dimensional biological ‘omic’ techniques, microbiome studies and drug levels, with potential for many more (Castle et al., 2004; Snowhite et al., 2002; Walter et al., 2011; Romero et al., 2006; Goldenberg et al., 2005; Amabebe et al., 2016; Kindinger et al., 2016; Parsons et al., 2014; Pereira et al., 2007; Price et al., 2011). In the context of pregnancy, the study of the FGF cytokine milieu has enabled the identification of IL-1β, IL-6, IL-8 and TNF-α as correlates of preterm delivery (Wei et al., 2010; Goldenberg et al., 2005; Tanaka et al., 1998; Discacciati et al., 2011; Goepfert et al., 2001; Jun et al., 2000).

Several systems have been developed to collect female genital tract secretions including: cervicovaginal lavage (CVL), polyester swabs, cervical wicks and ophthalmic sponges. Obstetric studies have traditionally used lavage for obtaining CVF samples or polyester swabs for CF collection however this produces large volumes of dilute CVF and small volumes of CF respectively (Walter et al., 2011; Pereira et al., 2007; Tanaka et al., 1998; Goepfert et al., 2001; Jun et al., 2000). Ophthalmic polyvinyl acetal (PVA) sponges are highly absorbent, have low binding affinity for protein and superior performance in recovery of cytokines from CF compared to other techniques (Lieberman et al., 2008). A novel technique using menstrual soft cups has emerged which enables larger volumes of undiluted CVF to be collected without a speculum and with the option of self-sampling (Boskey et al., 2003). There has been increasing use of this technique in the HIV-prevention field with excellent performance in quantifying immune mediators including cytokines (Archary et al., 2015; Price et al., 2011; Shukair et al., 2013; Cosgrove et al., 2016). To date neither ophthalmic sponges nor menstrual cups have been used in obstetric studies. The aim of this study was to evaluate and compare these methods for use in pregnant women. In addition, there is no consensus method for normalisation of cytokine concentration with some studies presenting data adjusted for sample dilution based on weight and some controlling for total protein (Castle et al., 2004; Dezzutti et al., 2011; Snowhite et al., 2002; Lieberman et al., 2008; Rohan et al., 2000; Quesnel et al., 1997; Marks et al., 2012). Here we present both methods and evaluate the effectiveness for each technique.

## 2 Methods and materials

### 2.1 Study subjects

Pregnant women attending routine antenatal booking appointments were invited to participate in this prospective observational study at St Mary's Hospital, London. This study was approved by the South East Coast RES Committee (13/LO/0107: The immunological basis of preterm delivery). Written informed consent was obtained enabling clinical data analysis and sample collection. Exclusion criteria were: multiple or in-vitro fertilization pregnancy, injecting drug use, HIV infection, current *Chlamydia trachomatis* or *Neisseria gonorrhoeae* infection.

### 2.2 Sample collection

Women were asked to provide EDTA whole blood and undergo FGF sampling by two methods at the three sequential mid trimester time points: 14–21, 22–25 and 26–31 weeks gestation (this sampling frame corresponds with routine antenatal clinic visits and the schedule of the parent study (13/LO/0107). Firstly, a speculum examination was performed enabling direct visualization of the cervical os to obtain a CF sample using an opthalmic PVA sponge (Eyetec™, Network Medical Ltd.), held in place for 1 min, with a sterile surgical needle holder. The PVA sponge was then replaced in a pre-weighed and labelled 15 mL sterile conical tube. A plastic loop was then used to obtain a smear from the lateral vaginal wall for microscopic and pH examination (Litmus test paper strip). Dry mounted glass slides were gram stained to enable evaluation of leucocyte cell count per high power field (in five cell increments), presence of *candida* and identify *Bacterial Vaginosis* by Hay Ison's Criteria (Ison & Hay, 2002; BASHH, 2012). Where clinical history indicated, *Chlamydia trachomatis* and *Neisseria gonorrhoeae* testing was performed (Nucleic acid amplification (AMPLICOR, Roche)). Women then underwent self or clinician insertion of a menstrual cup (MC) (Instead™ SoftCup, Evofem Ltd) to obtain cervicovaginal fluid (CVF). Correct vaginal placement of MC results in a lack of appreciation of the devise in situ, which was confirmed for each woman. The MC was removed at the end of the women's clinical appointment (minimum 5 min, maximum 30 min) and placed in a pre-weighed and labelled 50 mL sterile conical tube.

### 2.3 Specimen processing

EDTA whole blood was separated into plasma and peripheral blood mononuclear cells by sucrose density centrifugation within 4 h of sampling. Post collection weights were obtained for both CF swab and CVF cup containing conical tube tubes which were then placed on ice and stored at −80 °C within 4 h until ready for extraction.

### 2.4 CF

CF was extracted from the PVA sponge as previously described (Castle et al., 2004). To summarise, the PVA sponge was thawed on ice then 300 μL of extraction buffer (500 μL 1× protease cocktail I (Calbiochem®) + 10μl 10% Sodium Azide solution +0.75 g NaCl, final volume made to 50mls with 1× PBS solution, filter sterilised) was added to the PVA sponge in a Spin-x centrifuge filter unit (Costar, MA). This was spun at 13,000 rpm for 15 min at 4 °C, to elute the cytokines into the lower chamber. This process was repeated with another 300 μL of extraction buffer solution. The final 600 μL of CF was stored in 1.5 mL eppendorfs and frozen at −80 °C until further testing.

The dilution factor was calculated using the following formula: [(x − y) + 0.6 g of extraction buffer] / (x − y)], where x equals the weight of the PVA sponge + conical tube post collection and y is the weight of the PVA sponge and conical tube pre collection. The density of extraction buffer = 1.005 g/mL (Rohan et al., 2000).

### 2.5 CVF

The MCs were thawed for a maximum of 30 min on ice and centrifuged for 15 min at 400 g and 4 °C to pool the CVF at the bottom of the conical tube. The CVF (cells and supernatant) was divided into 100 μL aliquots into 1.5 mL eppendorfs using a positive displacement pipette (Rainin c10–100, Mettler Toledo). CVF was diluted 1 in 2 with the addition of 100 μL of extraction buffer (Castle et al., 2004; Cosgrove et al., 2016). The MC was re-spun if any CVF remained on the cup or in the conical centrifuge tube. The CVF was stored at −80 °C until further testing.

### 2.6 Cytokine measurement

Multi-spot chemi-luminescent assays (V-plex, Meso Scale Discovery® (MSD)) were used to measure 10 cytokines in plasma, CF and CVF (IFN-γ, IL-1β, IL-2, IL-4, IL-6, IL-8, IL-10, IL-12, IL-13 and TNF-α) according to the manufacturer's instructions. The sensitivity of these assays range from 0.02–938 pg/mL and 25 μL sample volume is required per reaction. Eluted CF samples, CVF samples (1 in 2 dilution) and neat plasma were analysed. All samples were run in duplicate. Matched plasma, CF and CVF samples were run on the same plate to limit any inter-plate variability. Data from the plates were analysed using MSD DISCOVERY WORKBENCH® version 4 and cytokine concentrations (pg/mL) were calculated using plate specific standard curves for individual cytokines. Where sample cytokine concentrations did not fall within the standard curve they were re run after dilution.

### 2.7 Protein measurement

Total protein concentrations in CF and CVF were estimated using the Bicinchoninic acid (BCA) method (Thermo Scientific ™ Pierce™). Samples were diluted 1 in 50 in extraction buffer and run in duplicate with a total volume per well of 25 μL. SoftMax Pro® version 5 was used to generate standard curves from which the protein concentration of each sample was interpolated (μg/mL).

### 2.8 Statistical analysis

Categorical variables were described in numbers and % and continuous variables summarised with median and interquartile ranges (IQR) and compared with the Kruskal–Wallis test. Cytokine concentrations were normalised to both sample dilution and total protein (expressed as a ratio of pg cytokine/mg protein). The correlation of cytokine concentration with specimen weight pre and post normalisation and associations with potential known confounders was calculated with Spearman's correlation co-efficient. Bonferroni correction was made for multiple comparisons (0.05/10) therefore a p value < 0.005 was deemed significant. Analyses were performed using SPSS (version 24).

## 3 Results

### 3.1 Study subjects

Between October 2013 and July 2014 20 women were recruited. One woman declined speculum examination and another withdrew from the study due to relocation. 53 samples collected from the remaining 18 women were included in the analysis. For patient demographics, see Table 1. Women were predominately Caucasian, married, nulliparous and non-smokers with healthy range BMIs.

**Table 1:** Participant characteristics and clinical data.

Characteristic	Value
Age (median years (IQR))	34.0 (30.0–34.0)
Ethnicity n(%)	
Caucasian	15 (83)
Black	0
Asian	2 (11)
Latin	1 (6)
Other	0
Relationship Status n(%)	
Married	14 (88)
Cohabiting	4 (22)
Single	0
BMI (median (IQR))	23 (20–24)
Smoking status n(%)	
Smoker	2 (22)
Non smoker	16 (88)
Parity (median (IQR))	0 (0–1)
Intercourse in preceding 24 h n	0
Practice of vaginal douching n	0
Vaginal pH	4.1 (3.8–4.6)
Bacterial Vaginosis	4/18 women in total^a^
current n	8/50 samples^b^
Candida	4/18 women in total
current n	5/50 samples
Preterm delivery n(%)	1 (5)

a These were different women to those in whom BV was identified.

b 50 corresponding vaginal microscopy samples were available.

Genital tract fluid weights and dilutions are shown in Table 2. CVF is a more viscous secretion than CF but can be easily handled with a positive displacement pipette. Eight times the amount of secretions were collected by the MC compared to PVA sponges and final dilutions were less using the MC. CVF: median 0.5 g, when diluted 1 in 2 with 100 μL extraction buffer produces approximately 5 × 200 μL aliquots, total 1000 μL versus CF: median 0.07 g, gives a 1 in 10 dilution [(0.07 + 0.6 g)/0.07 = 10] in 600 μL of extraction buffer. The MC method of CVF collection has a more constant dilution factor (2.0–10.0) compared to a wider range of dilution factors (1.3–61.0) observed with the process of elution of CF from the PVA sponge.

**Table 2:** Median specimen weight and dilution factors of genital tract secretions by sampling method.

Secretion type/method	Weight/g (IQR)	Range/g	Dilution factor (IQR)	Range of dilutions
CF by PVA sponge	0.07 (0.05–0.12)	0.01–0.54	9.6 (6.0–13.0)	1.3–61.0
CVF by MC	0.54 (0.35–0.82)	0.20–1.90	2.0 (2.0–4.0)	2.0–10.0

Anecdotally participants reported that they found both sampling methods acceptable but expressed that it would be advantageous if speculum examination were avoidable.

### 3.2 Collection method and sample volume

Genital tract fluid weights and dilutions are shown in Table 2. CVF is a more viscous secretion than CF but can be easily handled with a positive displacement pipette. Eight times the amount of secretions were collected by the MC compared to PVA sponges and final dilutions were less using the MC. CVF: median 0.5 g, when diluted 1 in 2 with 100 μL extraction buffer produces approximately 5 × 200 μL aliquots, total 1000 μL versus CF: median 0.07 g, gives a 1 in 10 dilution [(0.07 + 0.6 g)/0.07 = 10] in 600 μL of extraction buffer. The MC method of CVF collection has a more constant dilution factor (2.0–10.0) compared to a wider range of dilution factors (1.3–61.0) observed with the process of elution of CF from the PVA sponge.

Anecdotally participants reported that they found both sampling methods acceptable but expressed that it would be advantageous if speculum examination were avoidable.

### 3.3 Total protein

Total protein concentrations in undiluted CVF are higher than observed in CF (median CVF: 71731 μg/mL (IQR 56,489–109,967) versus CF: 19310 μg/mL (IQR 14,245–33,215), p < 0.0001).

### 3.4 Normalisation of cytokine concentrations

The effect of correcting female genital tract cytokine concentrations to sample dilution or normalising to total protein concentration is shown in Table 3, Table 4. CVF cytokine concentrations adjusted to either sample dilution or total protein were independent of sample weight with the exception of IL-1β and IL-6, however these associations with sample weight were removed by correction for multiple analyses. Conversely, CF cytokine concentrations were inversely associated with PVA sample weight after both dilution and total protein adjustment, see Table 4. From henceforth cytokine concentrations will be presented in pg/mL corrected for sample dilution.

**Table 3:** Effect of normalisation method on the correlation of CVF cytokine concentration and specimen weight.

Cytokine	Normalisation method	Median (IQR)	Spearmans (ρ)	P value
Pro-inflammatory				
IL-1β	As measured (pg/mL)	58 (20–443)	−0.401	0.004
	Dilution (pg/mL)	116 (44–1441)	−0.365	0.008
	Protein (pg/mg)	2 (1–14)	−0.351	0.013
IL-6	As measured (pg/mL)	8 (3–40)	−0.348	0.012
	Dilution (pg/mL)	24 (8–119)	−0.325	0.02
	Protein (pg/mg)	0.3 (0.1–1.6)	−0.359	0.01
IL-8	As measured (pg/mL)	621 (141–3170)	−0.281	0.046
	Dilution (pg/mL)	2636 (281–17,248)	−0.252	0.075
	Protein (pg/mg)	202 (36–453)	−0.264	0.067
TNF-α	As measured (pg/mL)	0.6 (0.2–1.6)	−0.166	0.243
	Dilution (pg/mL)	2 (0–5)	−0.168	0.238
	Protein (pg/mg)	0.02 (0.006–0.07)	−0.179	0.212

Immuno-regulatory				
IFN-γ	As measured (pg/mL)	3 (1–6)	−0.293	0.037
	Dilution (pg/mL)	6 (3−20)	−0.241	0.089
	Protein (pg/mg)	0.1 (0.04–0.3)	−0.217	0.130
IL-2	As measured (pg/mL)	0.5 (0.3–0.9)	−0.258	0.068
	Dilution (pg/mL)	1 (0.6–3.7)	−0.198	0.164
	Protein (pg/mg)	0.02 (0.006–0.04)	−0.252	0.077
IL-4	As measured (pg/mL)	0.1 (0.06–0.4)	−0.272	0.053
	Dilution (pg/mL)	0.4 (0.1–1.1)	−0.250	0.077
	Protein (pg/mg)	0.005 (0.002–0.11)	−0.208	0.147
IL-10	As measured (pg/mL)	0.7 (0.3–1.7)	−0.119	0.407
	Dilution (pg/mL)	3 (1–6)	−0.098	0.496
	Protein (pg/mg)	0.02 (0.01–0.09)	−0.119	0.411
IL-12	As measured (pg/mL)	0.2 (0.1–0.7)	−0.048	0.739
	Dilution (pg/mL)	0.6 (0.2–3.3)	−0.016	0.909
	Protein (pg/mg)	0.007 (0.003–0.05)	−0.042	0.770
IL-13	As measured (pg/mL)	4 (2–7)	−0.210	0.136
	Dilution (pg/mL)	12 (5–35)	−0.137	0.336
	Protein (pg/mg)	0.1 (0.05–0.4)	−0.193	0.180

**Table 4:** Effect of normalisation method on the correlation of CF cytokine concentration and specimen weight.

Cytokine	Normalisation method	Median (IQR)	Spearmans (ρ)	P value
Pro-inflammatory				
IL-1β	As measured (pg/mL)	35 (13–87)	−0.098	0.493
	Dilution (pg/mL)	1352 (178–5036)	−0.397	0.004
	Protein (pg/mg)	18 (6–36)	−0.285	0.045
IL-6	As measured (pg/mL)	5 (2−20)	−0.145	0.311
	Dilution (pg/mL)	252 (55–1375)	−0.370	0.008
	Protein (pg/mg)	3 (1−13)	−0.246	0.085
IL-8	As measured (pg/mL)	789 (421–1909)	−0.130	0.384
	Dilution (pg/mL)	34,491 (6553–119,938)	−0.496	0.0001
	Protein (pg/mg)	325 (155–812)	−0.123	0.397
TNF-α	As measured (pg/mL)	0.4 (0.2–1.0)	−0.029	0.841
	Dilution (pg/mL)	14 (4–60)	−0.601	0.0001
	Protein (pg/mg)	0.1 (0.1–0.5)	−0.448	0.001

Immuno-regulatory				
IFN-γ	As measured (pg/mL)	2 (1–4)	−0.214	0.132
	Dilution (pg/mL)	48 (9–327)	−0.391	0.005
	Protein (pg/mg)	1 (1–2)	0.426	0.002
IL-2	As measured (pg/mL)	0.4 (0.2–0.7)	−0.167	0.241
	Dilution (pg/mL)	9 (3–42)	−0.528	0.0001
	Protein (pg/mg)	0.2 (0.0–0.5)	−0.385	0.006
IL-4	As measured (pg/mL)	0.1 (0.0–0.2)	−0.210	0.139
	Dilution (pg/mL)	2 (0–6)	−0.333	0.057
	Protein (pg/mg)	0.04 (0.01–0.1)	−0.375	0.007
IL-10	As measured (pg/mL)	0.6 (0.4–1.6)	−0.409	0.003
	Dilution (pg/mL)	29 (6–87)	−0.574	0.0001
	Protein (pg/mg)	0.3 (0.1–1.0)	0.558	0.0001
IL-12	As measured (pg/mL)	0.3 (0.2–0.6)	−0.202	0.155
	Dilution (pg/mL)	10 (1–43)	−0.302	0.031
	Protein (pg/mg)	0.1 (0.06–0.3)	−0.368	0.09
IL-13	As measured (pg/mL)	5 (3−11)	−0.331	0.0018
	Dilution (pg/mL)	168 (48–636)	−0.471	0.0001
	Protein (pg/mg)	2 (1–5)	−0.538	0.0001

### 3.5 Cytokine concentrations by biological fluid type

**Fig. 1:**
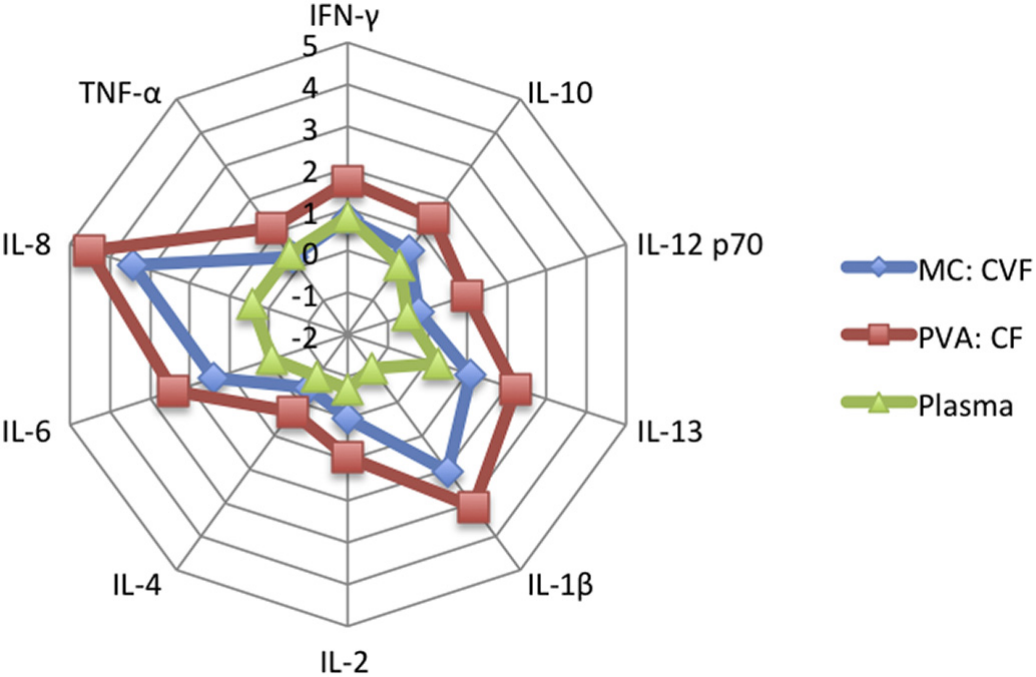
Spider chart to demonstrate log 10 median cytokine concentrations/pg/mL by biological fluid type.

All measured cytokines were detectable in both genital fluid types and plasma, see Fig. 1. The cytokine profile in CF and CVF displayed very similar ranking with high concentrations of pro-inflammatory cytokines: IL-8, IL-1β, IL-6 and TNF-α. Of the immune regulatory cytokines IL-13 was observed in the highest concentration in both genital tract fluids. For all cytokines median concentrations were 7–17 fold higher in CF compared to CVF, p < 0.0001.

Plasma cytokine concentrations were generally lower than those observed in female genital tract fluid (most notably pro-inflammatory cytokines: IL-8, IL-1β, IL-6 and immune regulatory: IL-13), see Fig. 1. Similar concentrations were observed between plasma and CVF for TNF-α and IFN-γ.

A similar percentage of plasma samples contained detectable cytokines compared to FGF samples, see Table 5.

**Table 5:** Comparison of cytokine detectability in genital tract fluid and plasma.

Cytokine (pg/mL)	PVA: Cervical Fluid	MC: Cervicovaginal Fluid	Plasma
	% Detectable	% Detectable	% Detectable
Pro-inflammatory			
IL-1β	100	100	100
IL-6	98	100	96
IL-8	100	100	100
TNF-α	96	94	100

Immuno-regulatory			
IFN-γ	96	100	100
IL-2	100	98	94
IL-4	88	90	92
IL-10	100	98	100
IL-12	90	85	88
IL-13	96	88	83

### 3.6 Effect of vaginal pH and leucocytes on cytokine concentrations

Correlations between cytokine concentration and vaginal pH were explored in both biological fluids. In CF, higher pH was positively correlated with all measured cytokines: IFN-γ (*r* = 0.414, p = 0.003); IL-10 (*r* = 0.387, p = 0.005); IL-12 (*r* = 0.360, p −0.009); IL-13 (*r* = 0.308, p = 0.028); IL-1β (r = 0.360, p = 0.009); IL-2 (*r* = 0.367, p = 0.008); IL-4 (*r* = 0.368, p = 0.008); IL-6 (*r* = 0.334, p = 0.017); IL-8 (*r* = 0.359, p = 0.013) and TNF-α (*r* = 0.329, p = 0.022). In CVF, IFN-γ (r = 0.329, p = 0.018) and IL-1β (*r* = 0.384, p = 0.005) were positively correlated with pH. After Bonferroni correction only CF IFN-γ, IL-10 and CVF IL-1β remained significantly positively associated with pH.

Next correlations between total leucocyte count per high power field on vaginal microscopy and FGF cytokine concentration were analysed. In CF all measured cytokines, with the except of IL-2, were positively correlated with increasing leucocyte count per high power field: IFN-γ (*r* = 0.358, p = 0.011); IL-4 (*r* = 0.382, p = 0.006); IL-10 (*r* = 0.331, p = 0.019); IL-12 (*r* = 0.373, p −0.008); IL-13 (*r* = 0.299, p = 0.035); IL-1β (*r* = 0.334, p = 0.009); IL-6 (*r* = 0.276, p = 0.05); IL-8 (*r* = 0.346, p = 0.019) and TNF-α (*r* = 0.302, p = 0.033). In CVF only IL-8 was significantly correlated with leucocyte count (*r* = 0.351, p = 0.012). There was a trend towards a positive correlation between leucocyte count and IFN-γ (*r* = 0.239, p = 0.094) and IL-1β (*r* = 0.266, p = 0.062). These associations did not withstand Bonferroni correction.

### 3.7 BV associated with higher median CF IL-1β, IL-13 and IFN-γ

The presence of BV was associated with higher concentrations of CF IL-1β (4117 pg/mL (IQR 1383–5364) vs 799 pg/mL (126–4222) p = 0.05) and IL-13 (367 pg/mL (IQR 252–782) vs 134 pg/mL (IQR 35–393) p = 0.04) compared to women with no BV, not withstanding Bonferroni correction. There was a trend towards a higher median IFN-γ in the FGF of women with BV compared to those without: CF 269 pg/mL (IQR 41–646) vs 27 pg/mL (IQR 8–275) p = 0.056; CVF 13 pg/mL (IQR 5–33) vs 4 pg/mL (IQR 2–15) p = 0.092.

### 3.8 Vaginal candida associated with higher median CF and CVF TH^1^ AND TH^2^ cytokines

The presence of vaginal candida was associated with higher CF concentrations of IFN-γ (558 pg/mL (IQR 266–83,308)) compared to women without candida (45 pg/mL (IQR 9–269)), p = 0.05, IL-2 (295 pg/mL (IQR 46–1682) vs 8 pg/mL (IQR 2–23)), p = 0.027, IL-12 (86 pg/mL (IQR 26–1420) vs 9 pg/mL (IQR 2–30)), p = 0.039 and TNF-α (122 pg/mL (IQR 36–2202) vs 13 pg/mL (IQR 4–43)), p = 0.05. A similar trend was seen for IL-13 (863 pg/mL (IQR 323–5328) vs 158 pg/mL (IQR 48–382)), p = 0.059 and IL-8 (295,306 pg/mL (IQR 58056–1,208,132) vs 29,999 pg/mL (IQR: 6617–70,560)), p = 0.077. These associations did not withstand Bonferroni correction.

In CVF, the presence of candida was associated with higher median cytokine concentrations of IL-12 (13 pg/mL (IQR 2–30)) compared to women with no candida (1 pg/mL (IQR 0–3)), p = 0.027. This trend was also observed between the presence of candida and IFN-γ (354 pg/mL (IQR 8–1674) vs 6 pg/mL (IQR 3–17)), p = 0.081, IL-1β (6322 pg/mL (IQR 515–11,086) vs 109 pg/mL (IQR 45–1055)), p = 0.081 and IL-8 (34,415 pg/mL (IQR 8968–36,420) vs 1870 pg/mL (IQR 296–8788)), p = 0.075. These associations did not withstand Bonferroni correction.

No significant differences in cytokine concentrations between second and third trimester time points were observed by either collection method, data not shown.

## 4 Discussion

In this study we found both PVA sponges and the menstrual cup to be valid methods of collection for the measurement of FGF cytokines with all the multiplex cytokines being measureable in each fluid. The highest concentrations of TH^1^ cytokines: IL-1β, IL-6, IL-8 and TNF-α observed in both fluid types are similar in profile to the literature for FGF from pregnant women (Kutteh & Franklin, 2001; Simhan et al., 2005; Nenadic & Pavlovic, 2008; Ryckman et al., 2009; Beigi et al., 2007).

CVF sampling by MC enables collection of high fluid volumes suitable for studies demanding multiple assays, with the opportunity to collect undiluted samples, making it useful for eliminating concerns about low-level contaminants/confounders that could be added during sample preparation. The option of self-sampling is also attractive and offers the potential for multiple sampling over pregnancy without the use of a speculum. Pregnant women in this study achieved comparable sample weights (median 0.5 g) to studies in non-pregnant women despite relatively short retention of the MC (Boskey et al., 2003). These figures add to the paucity of data on the optimal time for MC retention with one study in non-pregnant HIV-infected women demonstrating a median sample weight of 0.31 g with a minimum retention time of 60 min and another, in uninfected non-pregnant women, achieving a median sample weight of 0.5 g with a retention time of 5 s (Boskey et al., 2003; Jaumdally et al., 2017).

Conversely CF sampling by PVA swabs demonstrated the highest concentrations of cytokines. This reflects the fact that the cervix/fetal placental unit is the likely source of many of the cytokine producing cells and vaginal secretions dilute measurements in the CVF. However CF volumes are small and necessitate elution from the PVA sponge with extraction buffer introducing potential for confounding errors. Wider ranges of cytokine measurements were observed in the CF, which is likely to be the product of the variability in weight dependent dilution factors. Normalisation of CF cytokine concentrations to protein concentrations does not remove this weight dependent effect however CVF cytokine concentrations were largely independent of weight and protein concentration. The precision of volume dilution with CVF and reduced susceptibility to sample weight bias make this method advantageous.

The elevated concentrations of FGF pro-inflammatory cytokines: IFNγ and IL-12 observed in the presence of vaginal candidiasis mirror current knowledge of a IFN-γ CD4 TH^1^ adaptive and phagocytic candidacidal response induced by antigen presenting cells under the control of IL-12 and is in keeping with this inflammatory vaginitis (Romani, 1999; Yano et al., 2012). These data demonstrate the potential use of FGF cytokine measurement in detecting biological plausible mechanisms.

The observed association between FGF IL-1β and bacterial vaginosis has been described in the literature (Beigi et al., 2007; Sturm-Ramirez et al., 2000). Bacterial vaginosis is not believed to be an inflammatory condition yet is a known risk factor for preterm delivery. Whilst it is clear from these data that it does not appear to stimulate the same level of pro-inflammatory response in the genital tract of these pregnant women compared to candida, this shift in normal vaginal flora may generate some inflammation.

In addition to being the first to demonstrate the utility of these FGF collection methods in pregnant women, other strengths of this study include the evaluation of different normalisation methods (weight (based on accurate individual sample pre and post collection measurements) and protein concentration) and correlates of cytokines concentration with vaginal pH, leucocyte counts, bacterial vaginosis or candidiasis. This work also explored the changes in cytokine concentrations between the second and third trimester in the same individual.

Limitations of this work include the potential confounding in order of sampling in that collection of CF sample with the PVA sponge first may have impacted on subsequent collection of CVF with the MC, potentially reducing the measurable cytokine concentrations in the subsequent sample. In addition, not all women were tested for GC/CT testing with the potential for undiagnosed genital infection increasing cytokine concentrations however if this were the case, this would be observed across both methods. Some women preferred provider insertion of the MC and the data was not available to compare recovery between insertion techniques. The diagnoses of BV and candida were based on microscopy not culture and the strength of these correlates may be underestimated by the limits of the diagnostic criteria sensitivity. Although comparable to other work in this field, the relatively small sample size may have resulted in loss of statistically significant associations with clinical correlates through correction for multiple comparisons.

## 5 Conclusion

In summary both methods presented are acceptable and robust in pregnant women. The short collection time, self-insertion and large volumes of CVF collected by the menstrual cup are advantageous, however the choice of method also depends on the immune compartment of interest.

## Conflicts of interest

None.

## Sources

Wellcome Trust Clinical PhD Programme (WT102757/Z/13/Z).

NIHR Imperial Biomedical Research Centre (NG0215).
